# Crystal structures and calorimetry reveal catalytically relevant binding mode of coproporphyrin and coproheme in coproporphyrin ferrochelatase

**DOI:** 10.1111/febs.15164

**Published:** 2019-12-03

**Authors:** Stefan Hofbauer, Johannes Helm, Christian Obinger, Kristina Djinović-Carugo, Paul G. Furtmüller

**Affiliations:** 1 Department of Chemistry, Institute of Biochemistry, BOKU – University of Natural Resources and Life Sciences, Vienna, Austria; 2 Department of Structural and Computational Biology, Max Perutz Labs, University of Vienna, Austria; 3 Department of Biochemistry, Faculty of Chemistry and Chemical Technology, University of Ljubljana, Slovenia

**Keywords:** enzyme kinetics, ferrochelatase, heme biosynthesis, X-ray crystallography

## Abstract

Coproporphyrin ferrochelatases (CpfCs, EC 4.99.1.9) insert ferrous iron into coproporphyrin III yielding coproheme. CpfCs are utilized by prokaryotic, mainly monoderm (Gram-positive) bacteria within the recently detected coproporphyrin-dependent (CPD) heme biosynthesis pathway. Here, we present a comprehensive study on CpfC from *Listeria monocytogenes* (*Lm*CpfC) including the first crystal structure of a coproheme-bound CpfC. Comparison of crystal structures of apo-*Lm*CpfC and coproheme-*Lm*CpfC allowed identification of structural rearrangements and of amino acids involved in tetrapyrrole macrocycle and Fe^2+^ binding. Differential scanning calorimetry of apo-, coproporphyrin III-, and coproheme-*Lm*CpfC underline the pronounced noncovalent interaction of both coproporphyrin and coproheme with the protein (Δ*T*_m_ = 11 °C compared to apo-*Lm*CpfC), which includes the propionates (p2, p4, p6, p7) and the amino acids Arg29, Arg45, Tyr46, Ser53, and Tyr124. Furthermore, the thermodynamics and kinetics of coproporphyrin III and coproheme binding to apo-*Lm*CpfC is presented as well as the kinetics of insertion of ferrous iron into coproporphyrin III-*Lm*CpfC that immediately leads to formation of ferric coproheme-*Lm*CpfC (*k*_cat_/*K*_m_ = 4.7 × 10^5^ m^−1^·s^−1^). We compare the crystal structure of coproheme-*Lm*CpfC with available structures of CpfCs with artificial tetrapyrrole macrocycles and discuss our data on substrate binding, iron insertion and substrate release in the context of the CPD heme biosynthesis pathway.

**Enzyme:**

EC 4.99.1.9

**Database:**

pdb-codes of structural data in this work: 6RWV, 6SV3.

## Introduction

**Fig. 1:**
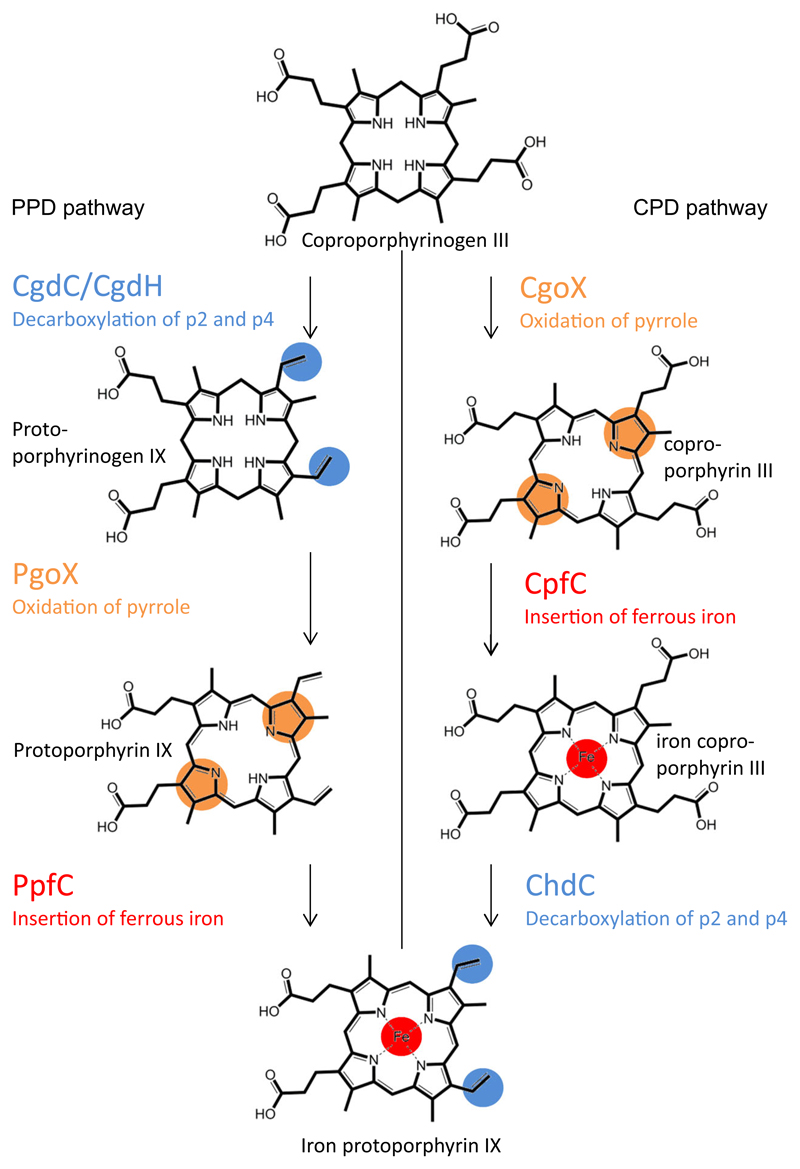
Overview of substrates and enzymes of the CPD and PPD heme biosynthesis pathways. Starting from the substrate coproporphyrinogen III prokaryotic heme biosynthesis takes two routes. (Left) The PPD pathway decarboxylates propionates at positions 2 and 4 (marked blue) by a radical mechanism mediated by SAM-enzyme coproporphyrinogen decarboxylase (CgdC)/coproporphyrinogen dehydrogenase (CgdH). Next, the pyrrole nitrogens are oxidized by PgoX (marked orange) and ferrous iron is inserted by PpfC (marked red); the final product is heme *b* (iron protoporphyrin IX). (Right) The CPD pathway first oxidizes pyrrole nitrogens by CgoX (homologous to PgoX; orange). Next, ferrous iron is inserted by CpfC (homologous to PpfC; red), and, finally, propionates at positions 2 and 4 are decarboxylated by ChdC (unique for this pathway, marked blue), thereby yielding heme *b*.

Knowledge of prokaryotic heme biosynthesis expanded rapidly by the discovery of the coproporphyrin-dependent (CPD) pathway, which is utilized by mainly Gram-positive bacteria [1]. This finding reopened many old questions that have to be readdressed and put new ones on the table. The CPD heme biosynthesis pathway differs from the protoporphyrin-dependent (PPD, formerly ‘classical’) pathway in the sequence of enzymatic reactions (Fig. 1) [1]. Processing of coproporphyrinogen to heme *b* takes three steps in both pathways, but in the CPD pathway decarboxylation of the propionate groups to the vinyl groups is the ultimate step and is catalyzed by the enzyme coproheme decarboxylase (ChdC) [2–5], which does not have an analogous enzyme in the PPD pathway. The other two enzymatic reactions, the oxidation of two pyrrole nitrogens and the insertion of ferrous iron into the porphyrin ring, are catalyzed by homologous enzymes in both pathways (Fig. 1): the protoporphyrinogen/coproporphyrinogen oxidase (PgoX, CgoX; formerly HemY) and the protoporphyrin/coproporphyrin ferrochelatase (PpfC, CpfC; formerly HemH) [1].

All studies investigating enzymes engaged in the CPD pathway, which were carried out prior to the discovery of this pathway, were performed in the belief that these enzymes were part of the PPD pathway, and therefore, protoporphyrinogen IX and protoporphyrin IX were used instead of coproporphyrinogen III and coproporphyrin III. While still a lot of valuable information is accurately given by these studies, concerning overall structure, identification of iron-binding, and porphyrin deprotonation site (conserved glutamate and histidine), information on this enzyme class with its endogenous substrate is still missing but is essential to understand substrate binding, conversion, release and regulation.

Chelatases in general are classified in two major classes (class I and II) and one minor class (for siroheme biosynthesis) [6,7]. Class I comprises ATP-dependent heteromeric complexes including magnesium chelatases (for chlorophyll and bacteriochlorophyll biosynthesis), aerobic cobalamin biosynthetic cobalt chelatases, and potentially nickel chelatases for coenzyme F_430_ synthesis [1]. CpfCs are members of class II chelatases, which are ATP-independent monomeric or dimeric enzymes. Other representatives of this class are sirohydrochlorin ferrochelatases, anaerobic cobalamin biosynthetic cobalt chelatases, and PpfCs [1].

The best-characterized ferrochelatase that inserts ferrous iron into coproporphyrin is from *Bacillus subtilis* (*Bs*CpfC) [8–11]. In this organism as well as in all Firmicutes, the enzyme is monomeric with a mass of about 35 kDa. By contrast, monomeric CpfCs (41 kDa) from Actinobacteria possess in addition [2Fe-2S] clusters of yet unknown function. The crystal structure of *Bs*CpfC with a single substrate iron bound in the active site exhibits iron coordination in a square pyramidal fashion by two amino acid residues, His183 and Glu264, and three water molecules. This iron was not present in the structure of a His183Ala variant, which strongly suggests that the insertion of a metal ion into coproporphyrin III occurs at this metal binding site. Structural studies of *Bs*CpfC in complex with *N*-methylmesoprophyrin (NMMP) and 2,4-disulfonic acid deuteroporphyrin IX (dSDP), which are similar to the substrate coproporphyrin III, are available [10,11]. The porphyrin macrocycles in none of these structures are positioned and oriented within active site as found in human ferrochelatases. Due to substantial structural differences between human PpfCs and prokaryotic, especially Firmicutes, CpfCs, it is not possible to draw mechanistic conclusions concerning catalytically relevant binding orientations for coproporphyrin III from available structural data since the substrate itself has two more propionates than protoporphyrin IX and therefore can interact to a much higher degree with the protein moiety [1]. The human ferrochelatase is a dimeric [2Fe-2S] cluster containing enzyme with distinct differences to CpfCs in the substrate/product binding site (e.g., a methionine residue coordinates the metal center after iron incorporation, in contrast to a tyrosine in monomeric CpfCs).

In order to understand the enzymatic mechanism of CpfCs, we combined biochemical and crystal structure analyses of CpfC from *Listeria monocytogenes* (*Lm*CpfC) in its apo-form and the product-bound state with iron coproporphyrin III (coproheme). This set of structures enables us to analyze the hydrogen bonding network of the active site, identifying relevant amino acid residues for coproporphyrin III (substrate) and coproheme (product) coordination. Subtle changes in secondary structure upon binding of the substrates are shown and analyzed in comparison with available data from CpfC from *B. subtilis* (*Bs*CpfC) in the apo-form and with bound inhibitors NMMP and dSDP [10,11]. The obtained data will be discussed to draw mechanistic conclusions.

## Results

### Biochemical analysis of apo-, coproporphyrin III-, and coproheme-*Lm*CpfC

**Fig. 2:**
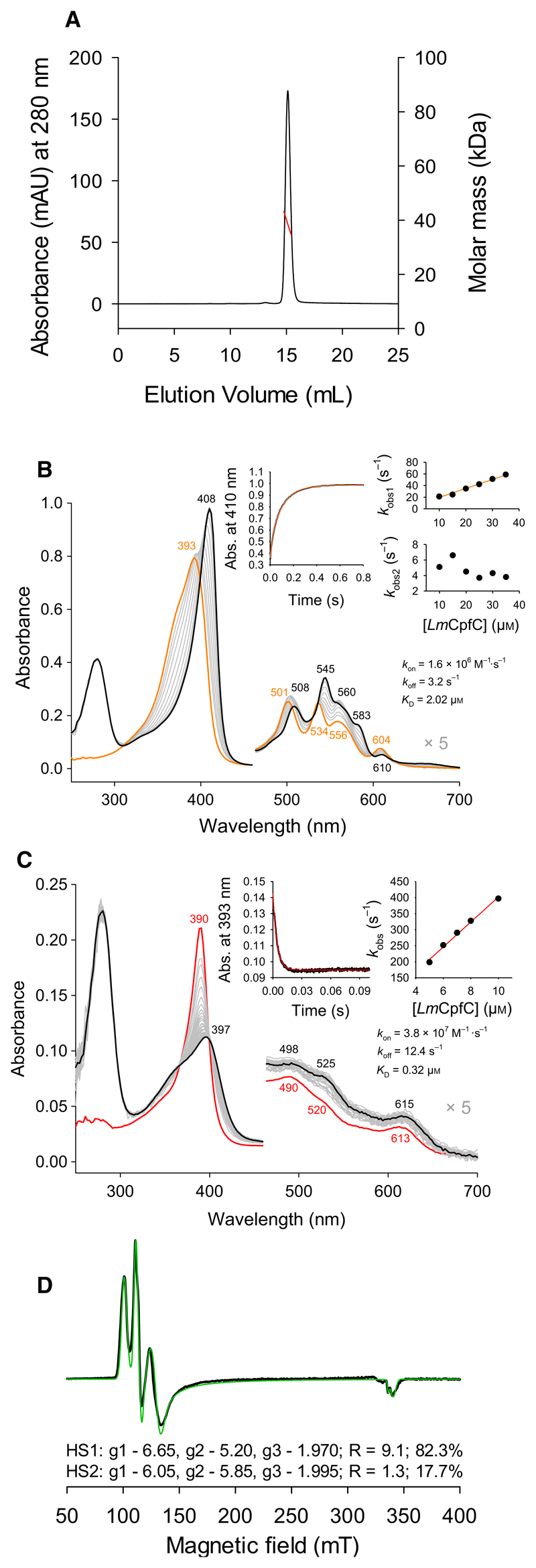
In solution studies of *Lm*CpfC in its resting state with substrate or product bound. (A) HPLC-SEC-MALS analysis of apo-*Lm*CpfC followed by UV-vis absorbance at 280 nm (black trace). Molar mass was determined by light scattering and is presented as a red line throughout the elution peak of the protein; buffer conditions: 1 × PBS + 200 mm NaCl. (B) Binding of 1 μm free coproporphyrin III (orange) to 10 μm *Lm*CpfC (final spectrum after 1 s in black), the insets show a time trace at 410 nm (black) with a double exponential fit (orange) and the dependence of *k*_obs1_ and *k*_obs2_ on increasing protein concentration of *Lm*CpfC; buffer conditions: 50 mm HEPES, pH 7.0. (C) Binding of 1 μm free coproheme (red) to 5 μm *Lm*CpfC (final spectrum after 0.1 s in black), the insets show a time trace at 393 nm (black) with a single exponential fit (red) and the dependence of *k*_obs_ to increasing protein concentration of *Lm*CpfC; buffer conditions: 50 mm HEPES, pH 7.0. (D) Low-temperature cw-EPR spectrum (black line) and simulation (green line) of coproheme-*Lm*CpfC; conditions: 50 mm HEPES, pH 7.0, 20% glycerol, 10 K, 2 mW, modulation amplitude: 1 mT, modulation frequency: 100 kHz. All measurements were performed at least in triplicates.

Expression and purification of *Lm*CpfC via metal chelate affinity chromatography using the C-terminal 6 × poly-His-tag yielded ~ 150 mg of purified protein per liter *Escherichia coli* culture. *Lm*CpfC was applied to analytical HPLC-SEC-multi-angle light scattering (MALS) analysis for control of monodispersity and molecular weight. The HPLC elution profiles of apo-*Lm*CpfC (Fig. 2A), coproporphyrin III-, and iron coproporphyrin III-*Lm*CpfC are identical. Recombinant *Lm*CpfC was a highly pure sample, with the monomeric peak accounting for more than 99% of the total area under all peaks. The determined mass by MALS was on average 38 kDa, which is well in agreement with the theoretical value of 37 kDa of the expressed construct (Fig. 2A).

UV-vis absorption spectroscopy of *Lm*CpfC with the substrate coproporphyrin III and the product coproheme is presented in Fig. 2B,C. The spectrum of coproporphyrin III-*Lm*CpfC is characterized by a Soret absorbance maximum at 408 nm and multiple bands in the visible region (508, 545, 560, 583, 610 nm), which are clearly shifted in comparison to the spectrum of free coproporphyrin III (Fig. 2B). Free coproporphyrin III or in complex with *Lm*CpfC is highly photosensitive, and therefore, all measurements were conducted in the dark.

Spectral transitions upon binding of coproporphyrin III to apo-*Lm*CpfC show a biphasic behavior (Fig. 2B). Time traces were fitted to a double exponential function, and the main absorbance change corresponds to *k*_obs1_, which linearly depends on the *Lm*CpfC concentration, resulting in *k*_on_ of 1.6 × 10^6^ m^−1^·s^−1^, *k*_off_ of 3.2 s^−1^, and *K*_D_ of 2.0 μm. By contrast, *k*_obs2_ (insets to Fig. 2B) does not depend on *Lm*CpfC concentration.

The spectrum of coproheme-*Lm*CpfC is characterized by a Soret maximum of 397 nm, Q-bands at 498 and 525 nm, and a charge transfer band at 615 nm (Fig. 2C) and is in part reminiscent (blue-shifted as compared the heme *b* proteins) to a six-coordinated high-spin (HS) [6-coordinated (6c)HS] His-Fe(III)-Tyr ligation [12]. This spectrum differs from the reported spectrum of coproheme-bound ChdC from *L. monocytogenes* (*Lm*ChdC), which has maxima at 393, 494, 538, and 630 [13]. Coproheme binding was monophasic and linearly depending on the *Lm*CpfC concentration (Fig. 2C, insets: *k*_on_ = 3.8 × 10^7^ m^−1^·s^−1^, *k*_off_ = 12.4 s^−1^, and *K*_D_ = 0.3 μm).

The coproheme iron in *Lm*ChdC was identified to be predominantly present in the rare 5-coordinated (5c)QS state, as was proven by the combination of resonance Raman (RR) and EPR spectroscopy [13]. This is not the case for coproheme in *Lm*CpfC, as simulation of the EPR spectrum shows species corresponding to a 5cHS (82.3 %) and a 6cHS (17.7 %) and no low-spin content (Fig. 2D).

**Fig. 3:**
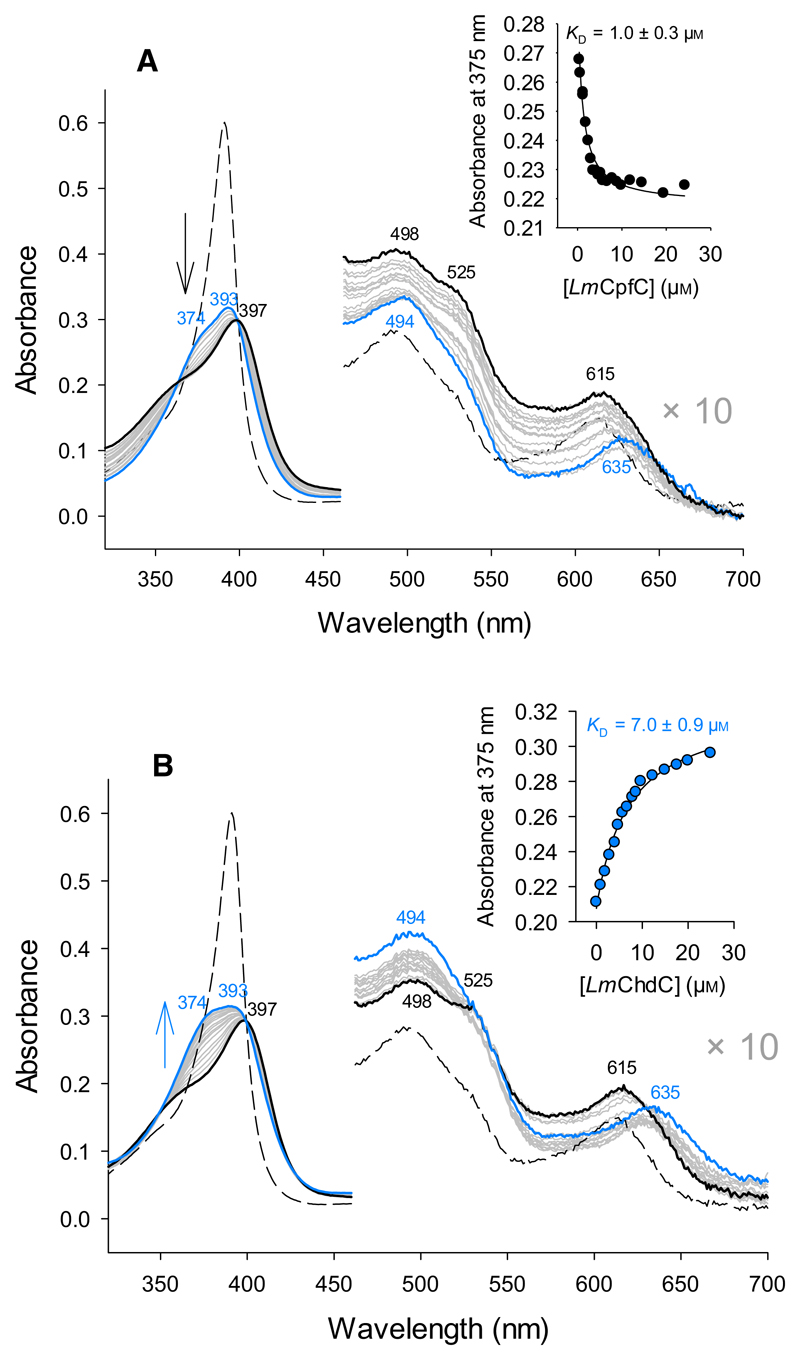
Coproheme binding to *Lm*CpfC and *Lm*ChdC. UV-vis spectra of (A) 5 μm preformed coproheme-*Lm*CpfC complex (black spectrum) and spectra during titration (gray) with *Lm*ChdC (up to 25 μm, final blue spectrum). The inset shows the absorbance at 375 nm plotted *versus Lm*ChdC concentration and a hyperbola fit. (B) 5 μm preformed coproheme-*Lm*ChdC complex (blue spectrum) titrated (gray) to yield the coproheme-*Lm*CpfC complex (25 μm, final black spectrum). Buffer condition: 50 mm HEPES, pH 7.0. All measurements were performed at least in triplicates.

Transfer of coproheme, which is the substrate for ChdC, was tested by addition of ChdC to the preformed coproheme-*Lm*CpfC complex and *vice versa*. Coproheme transfer works in both directions with a *K*_D_ of ~ 7 μm when *Lm*ChdC was added to coproheme-*Lm*CpfC and ~ 1 μm when *Lm*CpfC was added to coproheme-*Lm*ChdC (Fig. 3). This hierarchy agrees with coproporphyrin III binding studies of CpfC and ChdC from *Staphylococcus aureus* [14].

### Insertion of ferrous iron into coproporphyrin III mediated by *Lm*CpfC

**Fig. 4:**
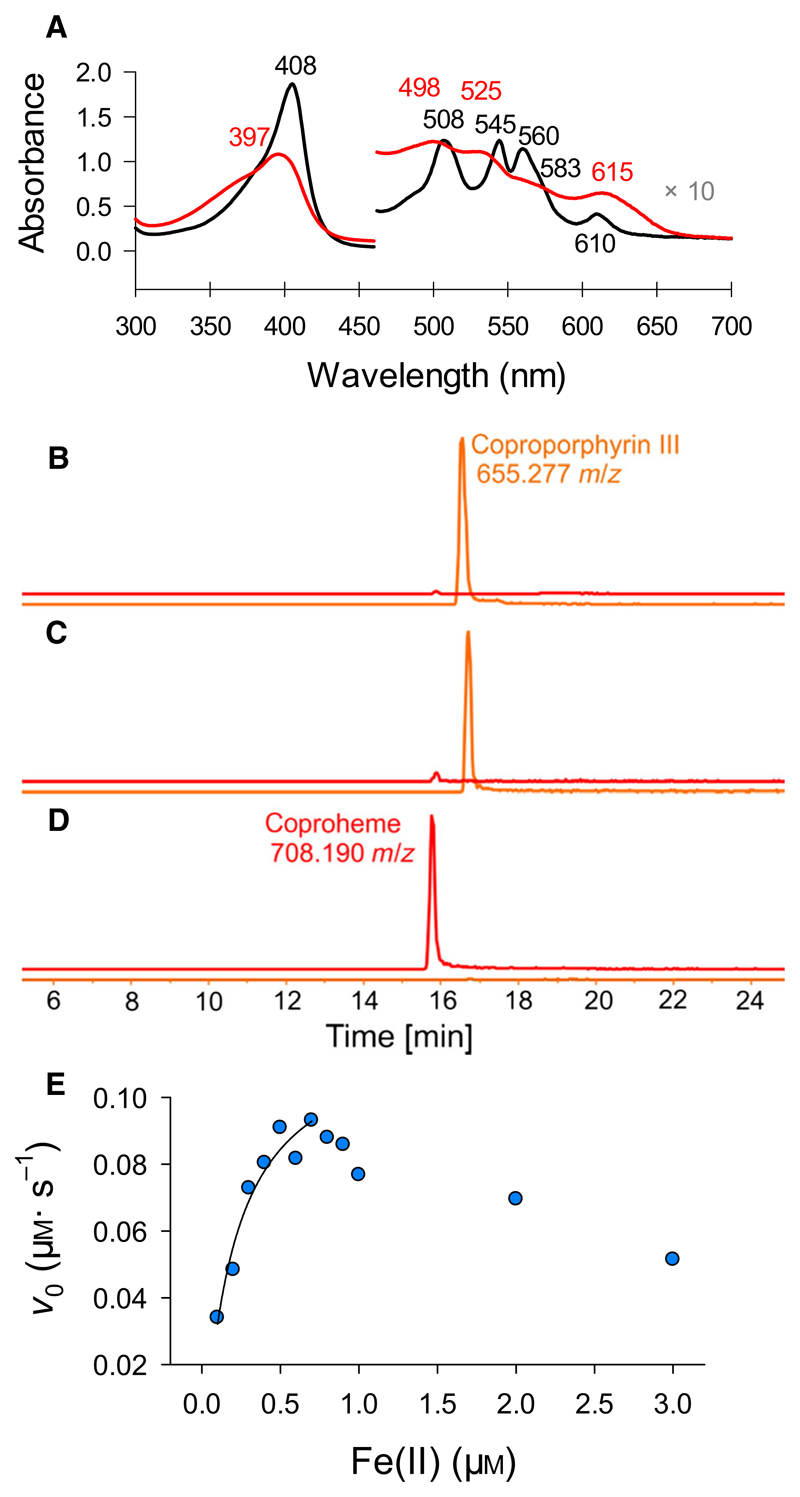
Spectral and kinetic studies on iron insertion into coproporphyrin III. (A) UV-vis absorption spectra of coproporphyrin III-*Lm*CpfC (black line) and after addition of ferrous iron (red line) in 50 mm HEPES pH 7.0 under anaerobic conditions and in the dark. (B) Mass spectrometric analysis of coproporphyrin III-*Lm*CpfC, the trace following the elution of the porphyrin substrate is depicted in orange, the trace for the porphyrin product coproheme in red. (C) Mass spectrometric analysis of coproporphyrin III and ferrous iron, (D) and of coproporphyrin III-*Lm*CpfC with ferrous iron. (E) Steady-state kinetics of insertion of ferrous iron into coproporphyrin III; conditions: 100 nm *Lm*CpfC, 10 μm coproporphyrin III, 0–3 μm Fe^2+^, 50 mm HEPES, pH 7.0, anaerobic under argon flow and in the dark. All measurements were performed at least in triplicates.

The capability of *Lm*CpfC to incorporate ferrous iron into coproporphyrin III was qualitatively tested by UV-vis spectroscopy, and the coproporphyrin III bound spectrum shifted toward the coproheme-bound spectrum upon addition of ferrous iron (Fig. 4A). Oxidation to ferric coproheme is instantaneous. Mass spectrometry of the exact same sample preparations, as for UV-vis spectroscopy, proved the insertion of ferrous iron into coproporphyrin III by *Lm*CpfC. The porphyrin substrate (655.28 Da) was the only detected porphyrin sample before the addition of Fe(II) (Fig. 4B, corresponding to black spectrum of Fig. 4A). Spontaneous insertion of ferrous iron into coproporphyrin III can be excluded, as is evident from Fig. 4C, where no *Lm*CpfC was added. When *Lm*CpfC was incubated with both substrates (i.e., Fe^2+^ and coproporphyrin III), coproheme (708.19 Da) was the only detected species, verifying the efficient enzymatic potential of this enzyme (Fig 4D, corresponding to red spectrum of Fig. 4A).

Steady-state kinetic parameters of the two-substrate reaction were derived for ferrous iron, which was varied in concentration, whereas coproporphyrin III was under saturated conditions. The catalytic specificity is comparable to the one previously reported for the highly similar CpfC from *S. aureus* (*Sa*CpfC) [15]. The determined *K*_M_ value is 0.28 μm for Fe(II), and *k*_cat_ is 7.9 min^−1^, yielding a catalytic specificity of 4.7 × 10^5^ m^-1^·s^−1^. Substrate inhibition was evident at Fe(II) concentrations higher than 0.8 μm, as for *Sa*CpfC [15] (Fig. 4E).

### Thermal stability of apo-, coproporphyrin III-, and coproheme-*Lm*CpfC

**Fig. 5:**
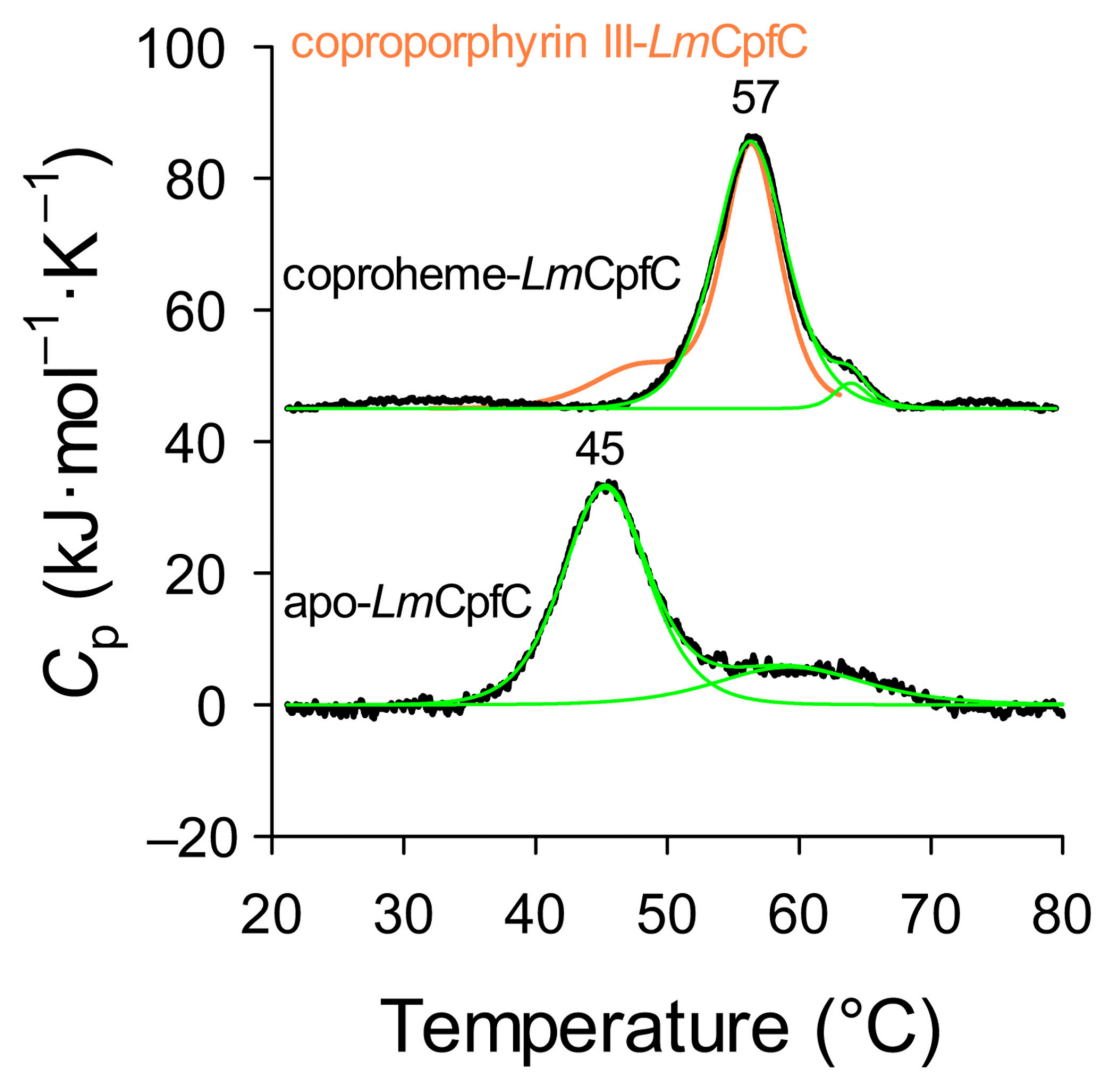
Thermal stability of apo-, coproporphyrin III-, and coproheme-*Lm*CpfC. DSC thermograms of apo- (lower black trace), coproheme-*Lm*CpfC (upper black trace) with the corresponding fits of the endotherms (in green) and coproporphyrin III-*Lm*CpfC (orange trace). The thermograms of coproheme*-Lm*CpfC and coproporphyrin III-*Lm*CpfC were shifted by 45 kJ mol^−1^·K^−1^ for better visibility; conditions: 14 μm protein, 50 mm HEPES, pH 7.0, heating rate: 60°C·h^−1^. All measurements were performed at least in triplicates

Differential scanning calorimetry (DSC) was performed to test the stabilizing effect upon substrate or product binding to *Lm*CpfC. Apo-*Lm*CpfC shows one main endotherm corresponding to a transition with a *T*_m_ value of 45 °C and van’t Hoff enthalpy/calorimetric enthalpy (Δ*H*_VH_/Δ*H*_cal_) ratio of 1.3, indicating cooperative unfolding of a monomer (Fig. 5), as expected based on the HPLC-MALS results (Fig. 2A). Coproporphyrin III-*Lm*CpfC (Fig. 5) and coproheme-*Lm*CpfC exhibit identical main transitions leading to a calculated *T*_m_ value of 57 °C and Δ*H*_VH_/Δ*H*_cal_ ratio of 0.8, also representing a cooperative unfolding behavior of a monomeric protein. The significant difference in *T*_m_ values of 11 °C suggests the pronounced formation of noncovalent bonds between the protein moiety and the macrocycles.

### Structures of apo- and coproheme*-Lm*CpfC

Diffraction data for apo- and coproheme-*Lm*CpfCs were collected to 1.64 Å resolution. Structures were solved with molecular replacement using *Bs*CpfC, the structurally best studied representative from Firmicutes ferrochelatases, as a search model [8], and refined to *R*_free_ values of 0.2013 (apo-*Lm*CpfC) and 0.2014 (coproheme-*Lm*CpfC); for complete data collection and refinement statistics, please see Table 1. The polypeptide is folded into a monomeric protein with two ferredoxin-like domains each with a four-stranded parallel β-sheet flanked by α-helices. Structural elements from both domains build up a cleft, which holds several amino acid residues that are conserved in ferrochelatases from different organisms [16]. This cleft was shown to be the binding site of NMMP) and 2,4,-disulfonic acid deuteroporphyrin IX (dSDP) [10,11].

**Fig. 6:**
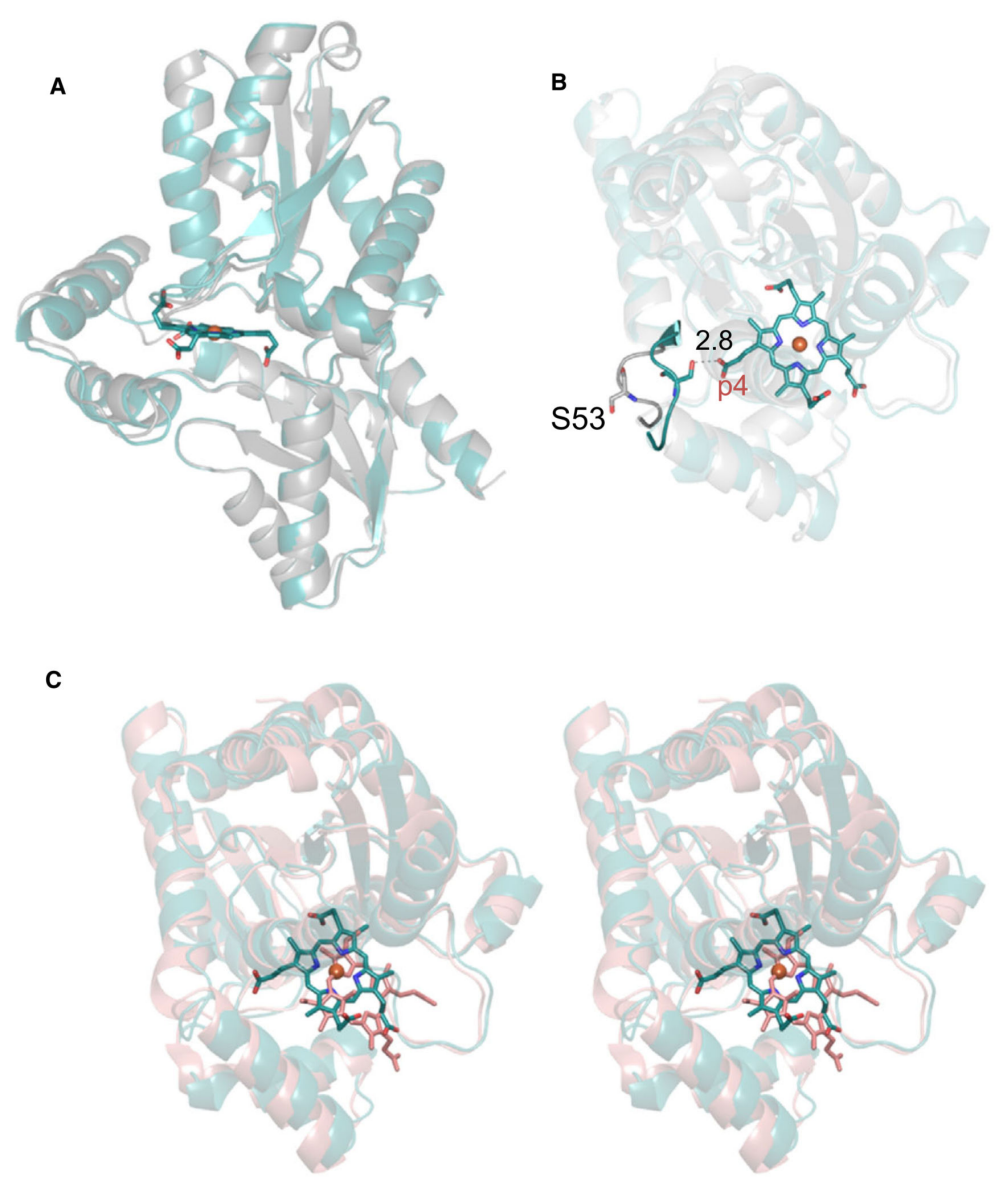
Overall structure of CpfC. (A) Overlay of apo- (gray, 6RWV) and coproheme-*Lm*CpfC (cyan, 6SV3). Secondary structural elements are represented as cartoon, coproheme as sticks, and the coproheme iron as orange sphere. (B) Top view of the overlay from (A) showing the S53 residues of both structures as sticks in the corresponding colors. (C) Stereo view of structural overlay of coproheme-*Lm*CpfC (cyan) and NMMP-bound *Bs*CpfC (pink, 1C1H); the ligands are shown as sticks. Figures were prepared with PyMOL (http://www.pymol.org).

**Fig. 7:**
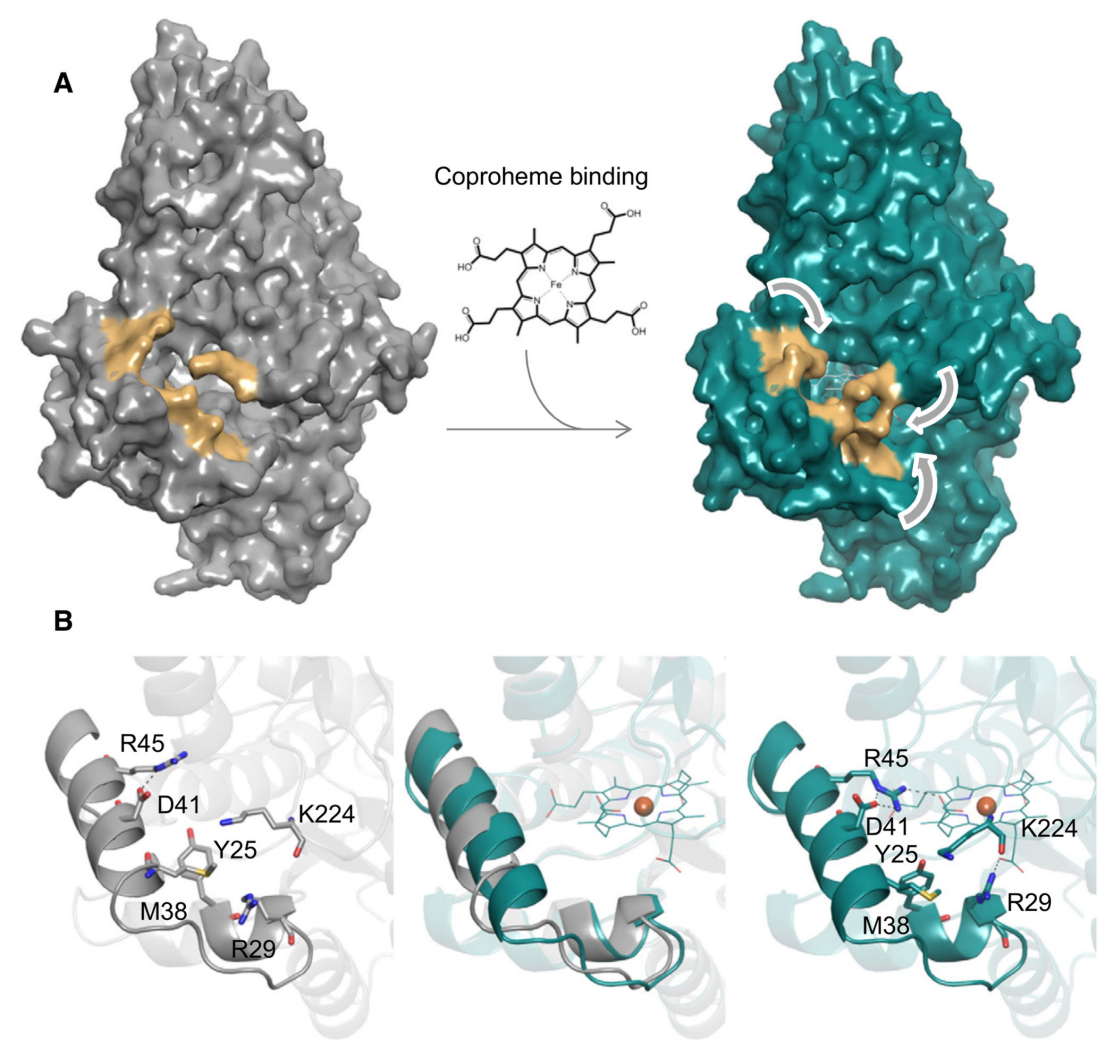
Structural effects of coproheme binding. (A) Apo-*Lm*CpfC (gray, left) and coproheme-*Lm*CpfC (cyan, right) are shown in a surface representation. Surfaces of residues Tyr25, Arg29, Met38, Asp41, Arg45, and Lys224 are presented in orange. (B) Positions of relevant amino acid residues (stick representation) and hydrogen bonding network of apo-*Lm*CpfC (left, gray) and coproheme-*Lm*CpfC (right, cyan) of α-helices (cartoon representation) that define the substrate excess channel. The middle panel shows an overlay of the aligned structures, coproheme is depicted as lines, and coproheme iron as orange sphere. Figures were prepared with PyMOL (http://www.pymol.org).

Structural comparison of apo- and coproheme-*Lm*CpfC shows that coproheme binding does not induce a notable conformational change in *Lm*CpfC (rmsd 0.297 Å) (Fig. 6A). Nevertheless, there is minor but evident structural rearrangement observed when apo- and coproheme-*Lm*CpfC are superimposed (Fig. 6B). One short loop hosting the Ser53 residue, which builds a hydrogen bond to the propionate group 4 (p4) of coproheme, undergoes a major change in orientation upon coproheme binding. In the apo-structure, the side chain of Ser53 is solvent exposed, while in the product-bound form, it points to the inner core of the protein, due to the interaction with p4 (Fig. 6B). Concomitantly, the two α-helices forming the substrate channel move closer to the inner core of the protein upon coproheme binding (Fig. 7A). The overall structure of coproheme-*Lm*CpfC is highly similar (rmsd 0.712 Å) to the NMMP-bound structure of *Bs*CpfC (1C1H) [11], though the porphyrin ring is positioned completely differently (Fig. 6C). Changes of the orientation of amino acid side chains upon coproheme binding are observed for Arg29, which forms a H-bond to p7, Met38 and Lys224, leading to closure of the binding cleft (Fig. 7). Similarly, Arg45 (H-bond to p6) adopts a notably different conformation in the apo-structure compared to the coproheme-bound structure, even though it is hydrogen bonded to Asp41 in both cases. The H-bonding pattern changes from one H-bond in the apo-form to a bidentate binding to the guanidinium group in the holo form (Fig. 7B).

Furthermore, coproheme binding induces a structural rearrangement of the two α-helices harboring Arg29 and Arg45, which manifests in a more compact overall fold (Fig. 7). CAVER [17] calculations of the putative substrate channels in apo- and coproheme-*Lm*CpfC show that the throughput (throughput values range from 0 to 1; the higher the value, the greater the importance of the tunnel) for the two calculated main channels in the apo-structure is higher than for the coproheme structure (apo: 0.867 and 0.799; coproheme: 0.832 and 0.757). Additionally, the bottleneck radii for channels in apo-*Lm*CpfC are larger (2.6 and 2.3 Å) than in coproheme-*Lm*CpfC (2.2 and 2.0 Å).

**Fig. 8:**
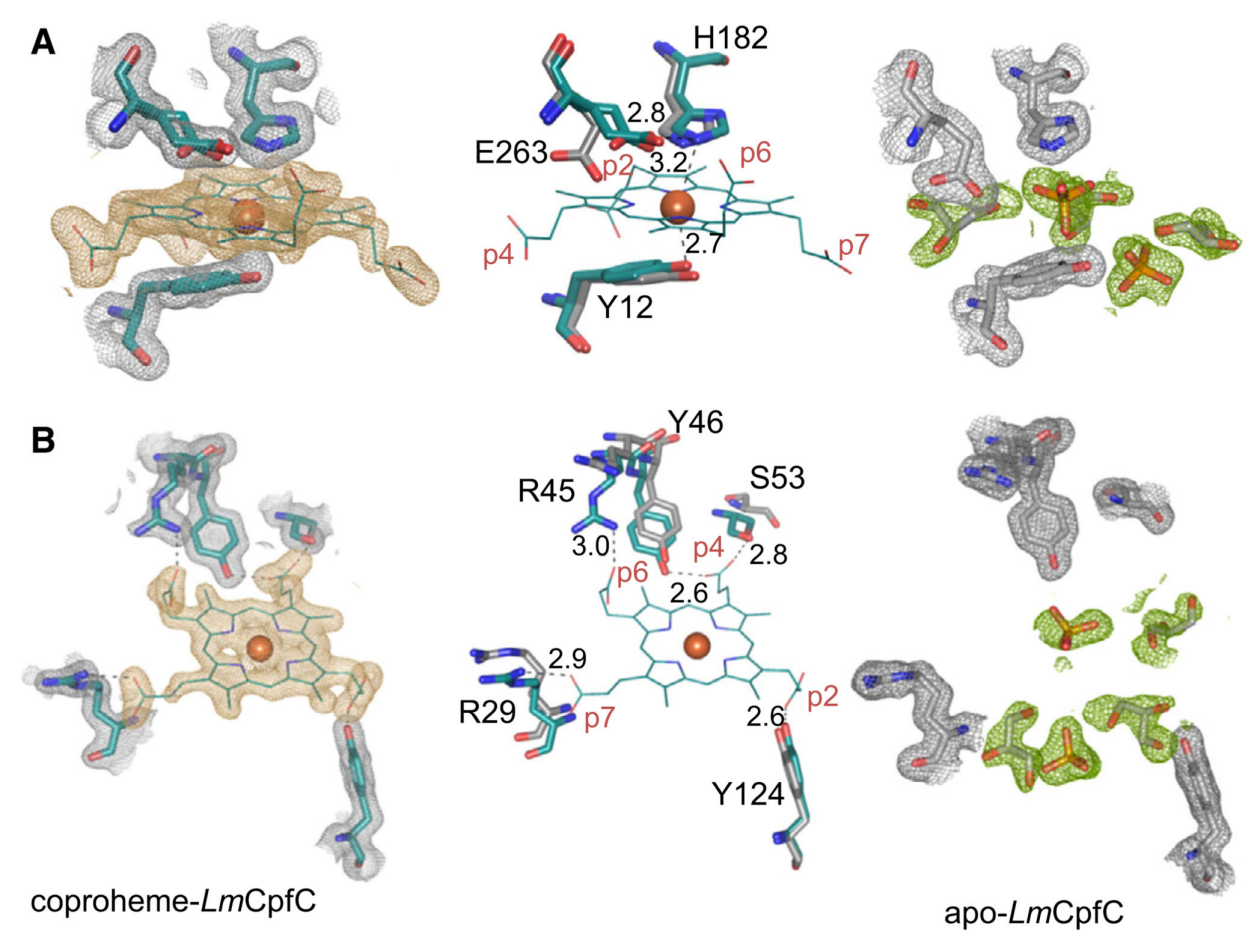
Substrate/product binding site of *Lm*CpfC. (A) Presentation of the distal and proximal active site architecture in coproheme-*Lm*CpfC (cyan, 6SV3, left panel), apo-*Lm*CpfC (gray, 6RWV, right panel), and the respective overlay (middle panel). Coproheme is depicted as lines, the coproheme iron as orange sphere, amino acids as sticks, solvent molecules occupying the active site of apo-*Lm*CpfC are shown as orange sticks. Composite (2*F*_o_ − *F*_c_) electron density maps are represented for all modeled structural elements (contoured at 1 σ); gray for all protein amino acid residues, orange for the coproheme itself, and green for solvent molecules in the apo-structure. (B) Presentation of noncovalent interactions of coproheme with the protein the same color code as in (A). Distances, representing potential H-bonding interactions, are shown as black dashed lines. Figures were prepared with PyMOL (http://www.pymol.org).

Architecture of the active site of ferrochelatases is described by the proximal Tyr12 and the distal His182/Glu263 pair (Fig. 8A), which has been shown to be the metal binding site of CpfCs [8,15]. In the given structure, Glu263 was best modeled in a double conformation, forming an H-bond in one conformation with His182; this reflects the flexibility of this residue (Fig. 8A, left panel). In the apo-structure, the substrate/product binding site is occupied by solvent molecules (phosphates and glycerols). Interestingly, the orientation of Glu263 is notably different compared to the product bound, pointing away from the His182 (Fig. 8A, right panel). The distance of His182 to the coproheme iron is 3.2 Å (Fig. 8A, middle panel), which is a little bit too far for pure 6cHS species, as it is reflected in solution data obtained by low-temperature EPR spectroscopy (Fig. 2D).

Figure 8B shows the coordination of coproheme in the binding cleft of *Lm*CpfC. The electron densities allow assignment of the respective propionates without doubt, as coproheme is an asymmetric porphyrin. Tyr124 hydrogen bonds to p2, whereas p4 is, as mentioned above, hydrogen bonded to Ser53 and to Tyr46. Propionate 6 (p6) and p7 form salt bridges to positively charged Arg residues (Arg 45—p6; Arg29—p7). The positions of Arg29 and Arg45 differ significantly in the apo- and the coproheme-*Lm*CpfC structure, as described above (Fig. 7B), while Tyr124 and Tyr46 overlay nicely (Fig. 8B). The rearrangement of Arg29 and Arg45, already mentioned above, is involved in the modulation of the substrate/product binding cleft (Fig. 7).

**Fig. 9:**
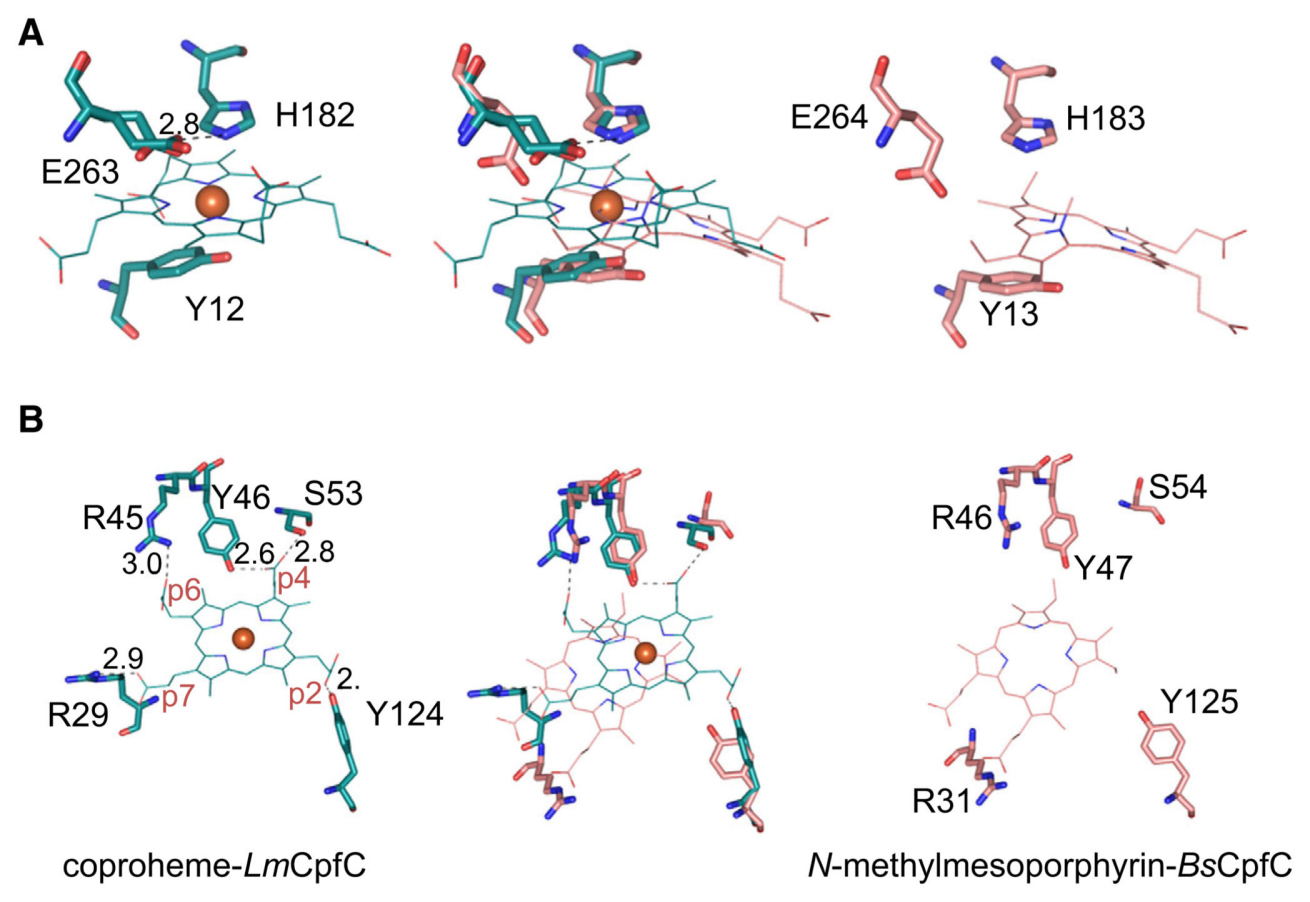
Porphyrin binding positions in Firmicutes CpfCs. (A) Presentation of distal and proximal active site architecture of coproheme-*Lm*CpfC (cyan, 6SV3, left panel), NMMP-*Bs*CpfC (pink, right panel, 1C1H), and the respective overlay (middle panel). (B) Presentation of noncovalent interactions of coproheme with the protein using the same color code as in (A), NMMP is shown as pink lines. Distances representing H-bonding interactions are shown as black dashed lines. Figures were prepared with PyMOL (http://www.pymol.org).

Comparison of the coproheme-bound structure of *Lm*CpfC with NMMP and dSDP bound structures of *Bs*CpfC (pdb-codes: 2Q3J, 2Q2N, and 1C1H) [10,11] clearly shows that the porphyrins are positioned substantially differently in respective structures (Fig. 9). In Fig. 9, the coproheme-bound *Lm*CpfC structure is compared to the NMMP-bound *Bs*CpfC; dSDP bound to wild-type *Bs*CpfC is even more surface exposed and further away from the coproheme binding position (not shown). While the proximal Tyr12 (Tyr13 in *Bs*CpfC) is indifferent (Fig. 9A), the p2-coordinating Tyr, the p4-coordinating Ser, and the p7-coordinating Arg are differently oriented in the respective structures (Fig. 9B). With the present coproheme-*Lm*CpfC structure, we are confident to describe the catalytically relevant binding orientation of coproheme and proposedly also of the substrate coproporphyrin III.

## Discussion

The enzymatic reaction of insertion of ferrous iron into coproporphyrin III by CpfC is only described for a few cases [5,15], since the CPD pathway was only identified in 2015 [2]. Earlier studies on ferrochelatases from Gram-positive organisms were performed using protoporphyrin IX as substrate for iron insertion [8–11].

**Fig. 10:**
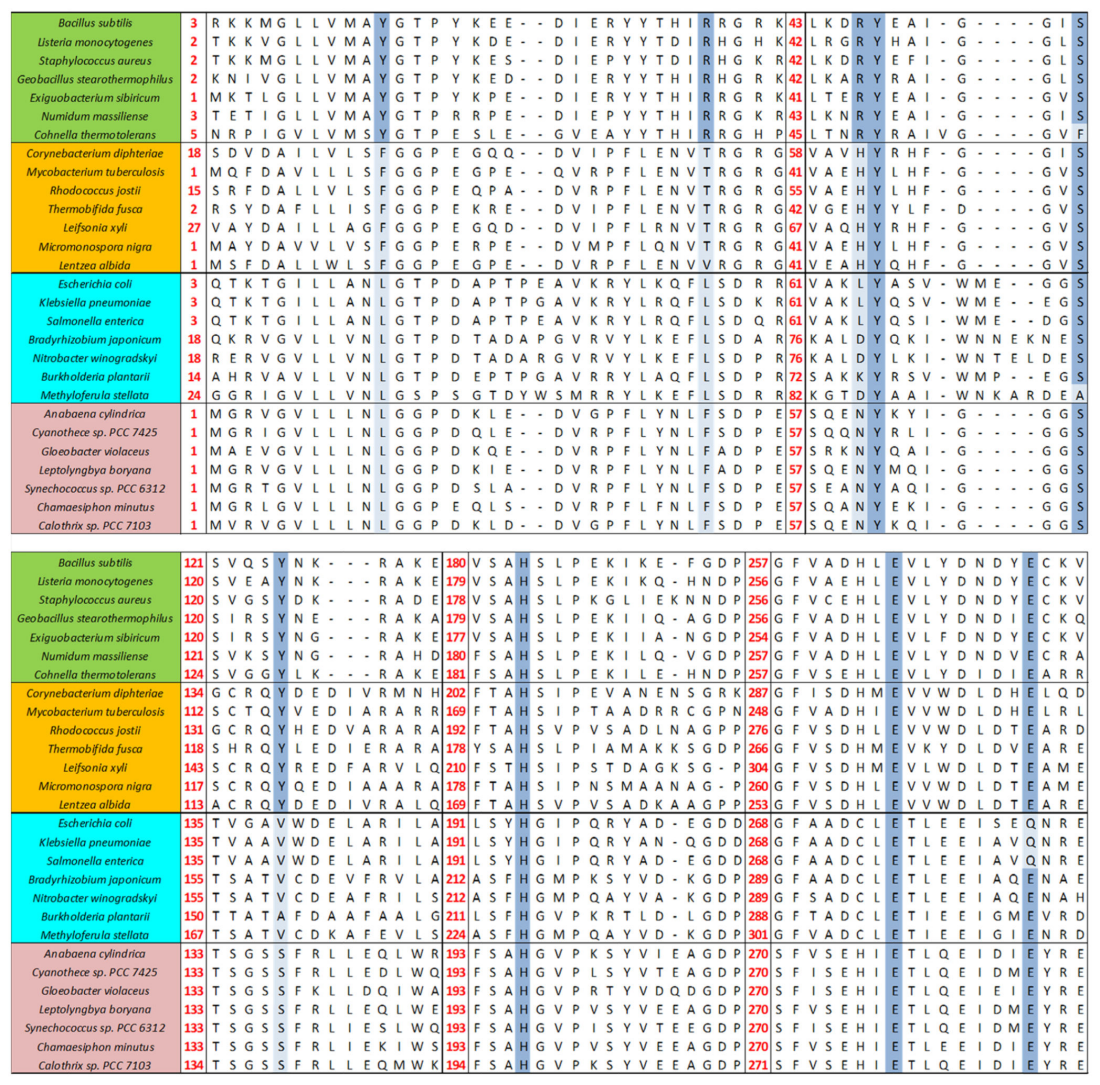
Sequence alignment of CpfCs and PpfCs. Firmicutes are blocked and marked in green, Actinobacteria in orange, Proteobacteria in cyan, Cyanobacteria in pink. Positions of relevant amino acids are marked in dark blue (conserved based on Firmicutes CpfCs) and light blue (not conserved with regard to Firmicutes CpfCs). The alignment was calculated using the program MUSCLE [47] part of the MEGA 5 package [48]. Accession numbers: WP_087991254.1, WP_010989926.1, WP_029550342.1, WP_026829087.1, WP_033013831.1, WP_054950580.1, WP_003851538.1, WP_027091271.1, WP_060815770.1, WP_011598893.1, WP_011292369.1, WP_043993400.1, WP_091076058.1, WP_089912683.1, WP_042973710.1, WP_064162154.1, WP_042310037.1, WP_028173852.1, WP_041345393.1, WP_083931451.1, WP_042623923.1, WP_012629873.1, WP_015216662.1, WP_011140841.1, WP_017289411.1, WP_015123173.1, WP_019488648.1, WP_015160918.1.

**Fig. 11:**
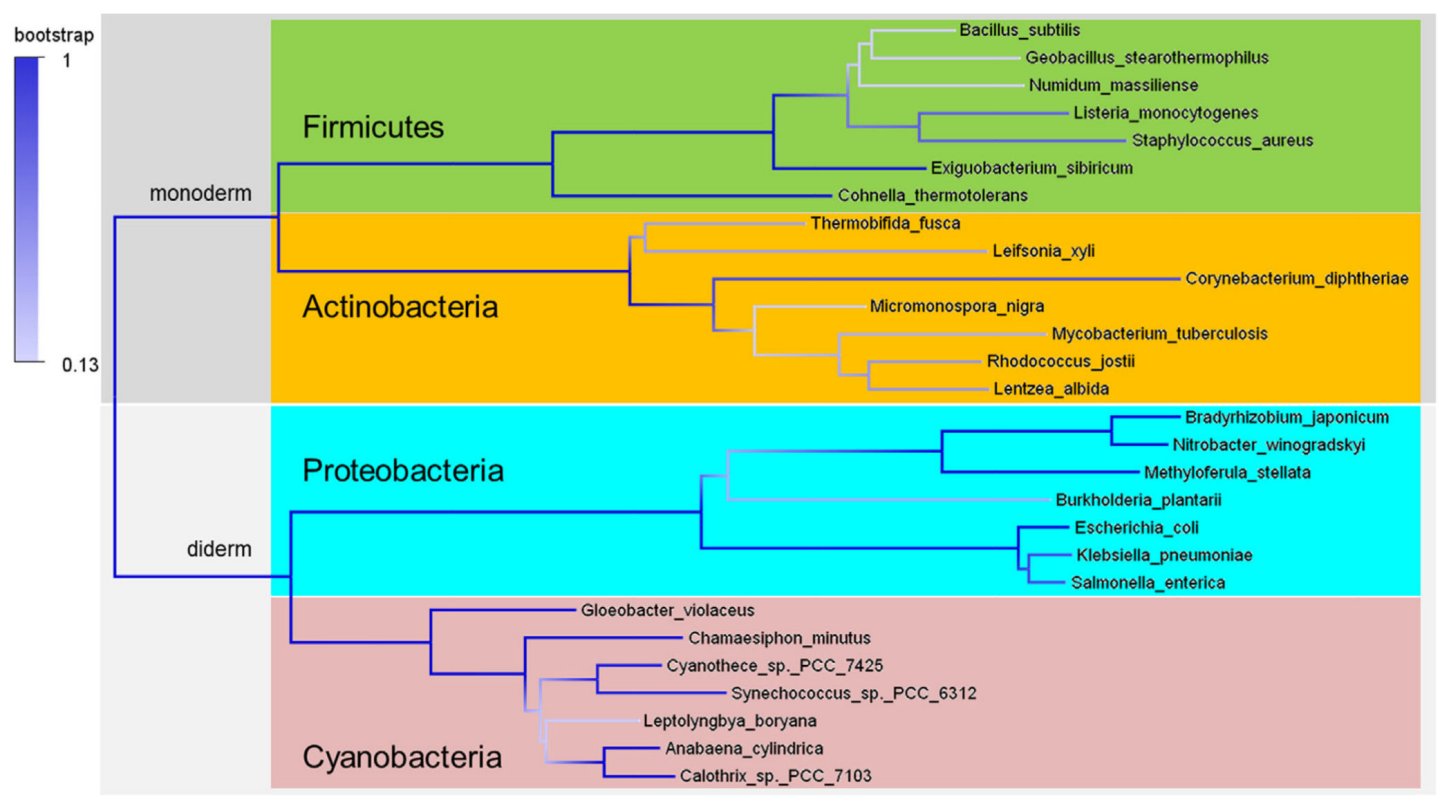
Phylogenetic tree. Phylogenetic analysis (maximum likelihood) of CpfCs (monoderm) and PpfCs (diderm) based on the sequence alignment presented in Fig. 10. Proteins from Firmicutes, Actinobacteria, Proteobacteria, and Cyanobacteria are highlighted in green, orange, cyan, and pink, respectively.

The catalytic efficiency of *Lm*CpfC for insertion of ferrous iron is very similar to the one reported for *Sa*CpfC [15] (Fig. 4). This is not surprising, as the sequence of these ferrochelatases is highly conserved, the sequence identity and similarity within CpfCs from Firmicutes is high (Fig. 10). Further, actinobacterial CpfCs, having a C-terminal [2Fe2S] cluster of unknown function, have their own phylogenetic clade and monoderm (Gram-positive) representatives clearly distinguish themselves phylogenetically from diderm (Gram-negative) representatives, as they can be rooted against each other (Fig. 11). Because of this sequence conservation within Firmicutes, the enzyme kinetics, spectral and structural results, presented in this work for *Lm*CpfC, can be assumed to be valid for the entire phylogenetic clade of CpfC.

**Fig. 12:**
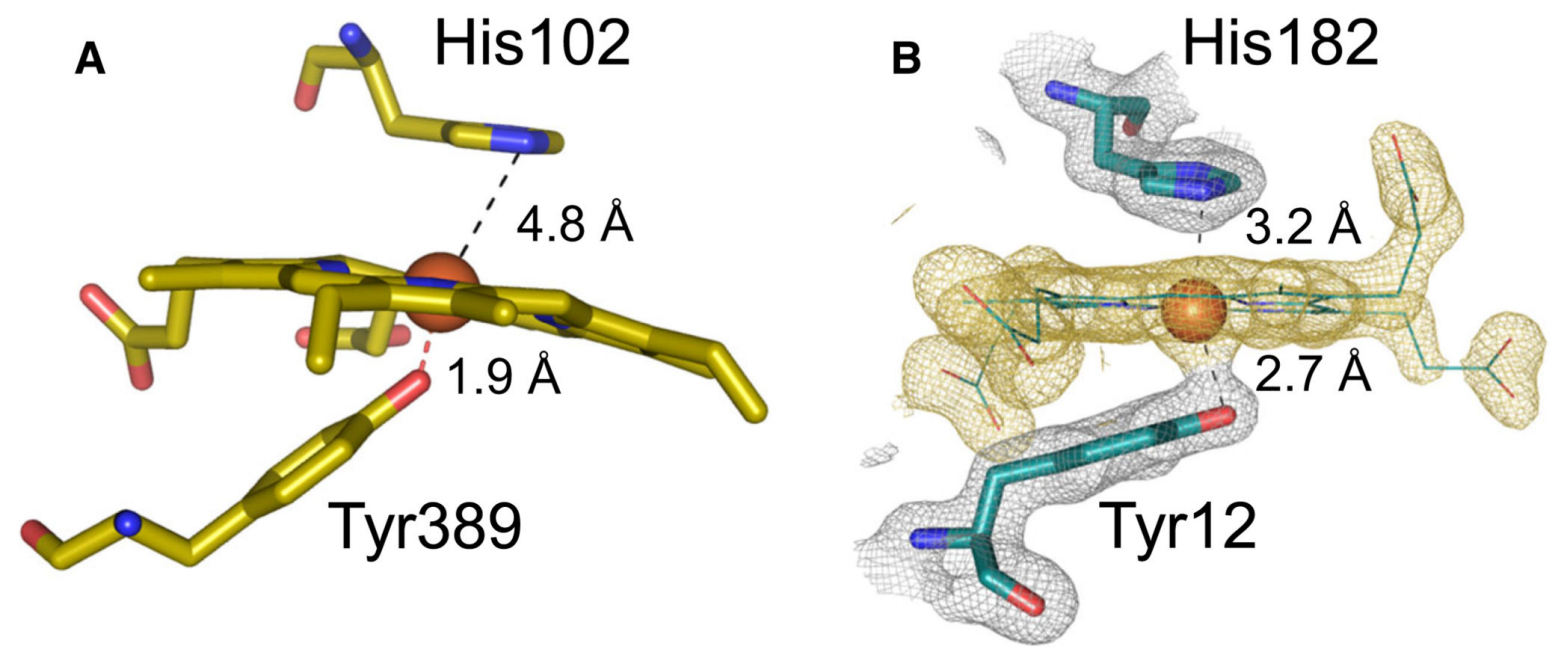
Active sites of catalase and CpfC. (A) Heme environment of catalase from *Neurospora crassa* (pdb-code: 3EJ6) showing the distal histidine and the proximal tyrosine residue. (B) Heme environment of coproheme-*Lm*CpfC (6SV3) with electron densities (contour 1 σ). Figures were prepared with PyMOL (http://www.pymol.org).

Spectral characterization of coproheme-*Lm*CpfC demonstrates that the ferric coproheme iron is completely HS (Fig. 2). As stated above, the UV-vis spectral signatures are reminiscent of a six-coordinated HS (6cHS) His-Fe(III)-Tyr ligation [12], but blue-shifted in comparison to heme *b* bound proteins, due to the missing vinyl conjugation [18]. When taking the EPR spectrum into account, it becomes obvious that the main HS signal represents a rhombic 5cHS coproheme iron, accounting for more than 80% of the signal intensity, with no distal ligand. The minor species, accounting to < 20% of the EPR signal, is significantly more axial and indicates a 6cHS state, with His182 being most probably the distal ligand (Fig. 2D). The EPR data are in agreement with the distance of 3.2 Å between the coproheme iron and His 182 (Figs 8A and 12B), which is too long to cause a 6cHS. In solution, the distances vary due to the dynamics of the system, and this leads to the minor 6cHS species.

Binding of coproporphyrin III or coproheme to apo-*Lm*CpfC has a strong stabilizing effect on the protein, as reflected in the increased thermal stability of the resulting complexes (Fig. 5). In the apo-protein, the active site (i.e., coproporphyrin III binding site) is filled with solvent molecules that are released upon binding of the macrocycle as seen in the crystal structures of apo-*Lm*CpfC and coproheme-*Lm*CpfC (Fig. 8). Each propionate group of coproheme forms one (p2, p6, p7) or two (p4) hydrogen bonds or salt bridges to the protein moiety, explaining the increase in thermostability by tying together secondary structural elements, which are far apart from each other. Since the increase in *T*_m_ is similar in coproporphyrin III-*Lm*CpfC and coproheme-*Lm*CpfC, it is reasonable to assume that the propionate groups and the protein exhibit the same interactions in the two complexes.

Out of the five identified residues (Tyr124—p2; Ser53/Tyr46—p4; Arg45—p6; Arg29—p7), only serine (Ser54 in *Bs*CpfC) has a documented effect for porphyrin interaction [9]. It has been suggested to be part of a docking site for a protein with the function of delivering any of the two substrates (coproporphyrin III and Fe^2+^), or retrieving the (copro)heme product [9]. Consequently, the proximal and axial ligation (by Tyr12 and partially by His182) of the coproheme iron does not have a pronounced contribution to stabilization of the coproheme-*Lm*CpfC. Binding of coproheme has a strong stabilizing effect on ChdC, for which coproheme is the substrate and redox cofactor. In *Sa*ChdC, the melting temperature rises by 14 °C (59–73 °C) and in *Lm*ChdC by 20 °C (35–55 °C) compared to the respective apo-proteins, whereas heme *b* bound ChdCs show the same unfolding behavior as the apo-protein [19]. Therefore, the stabilization is only due to the additional interaction of p2 and p4, and in *Lm*ChdC, a pronounced H-bonding network between coproheme and the respective amino acid side chains is established, spanning from p2 to p4 [20,21].

The increase in thermostability is due to coproheme binding, as H-bonding interactions between propionates and residues Arg29, Arg45, Tyr46, Ser53, and Tyr124 are established and a structural rearrangement is induced which leads to a more compact subunit architecture of the coproheme-bound *Lm*CpfC. Further, *Lm*CpfC structures superimpose well with other structures of Firmicutes CpfCs from other organisms (Fig. 6). The substrate/product is bound in the cleft between the two ferredoxin-like domains.

A closer look into the active site of coproheme-*Lm*CpfC reveals that amino acid residues are present, which are well known from heme centers in peroxidases and catalases. A proximal tyrosine residue and a distal histidine are present in heme catalases [22]. Still, in catalases the distal histidine interacts via a water molecule with an asparagine residue and the distance to the heme iron is significantly longer; ~ 4.5–5 Å [22–25]. In coproheme*-Lm*CpfC, the distal histidine (His182) is H-bonded to Glu263, which form the amino acid pair, essential for metal binding and porphyrin deprotonation prior to ferrous iron insertion [26]. Therefore, the distal histidine is arrested and not able to deprotonate an incoming hydrogen peroxide molecule. This is necessary, since the coproporphyrin III/coproheme binding site is highly accessible in order to bind the substrate and release the product for further catalysis by ChdC. The proximal tyrosine is closer to the iron in catalases (1.9 Å) (Fig. 12A) compared to Tyr12 in coproheme-*Lm*CpfC (2.7 Å). Also, the relative position of the proximal tyrosine differs significantly in both systems, while in ferrochelatase the tyrosine is positioned almost parallel to the porphyrin ring, with a potential π-stacking interaction with pyrrole ring A (Figs 8 and 12), the proximal tyrosine in catalases is positioned in an ~ 45° angle to the heme plane (Fig. 12) and is H-bonded to a neighboring arginine residue [22].

The inability to form Compound I upon addition of hydrogen peroxide, due to the structural restraints of iron ligation discussed above, is the reason that no catalase and no ChdC activity is detected in *Lm*CpfC. Interestingly, p2 in coproheme-*Lm*CpfC is in close proximity to a tyrosine residue (Y124), similar to Firmicutes ChdCs, where p2 is interacting with a catalytically relevant essential tyrosine (radical site), which triggers decarboxylation of p2 and later p4 [3,21,27,28]. The inactivity toward peroxides is a necessity, since the only task of a ferrochelatase is to insert ferrous iron into a porphyrin. Once coproporphyrin is loaded with iron, it becomes an active redox cofactor that has to be regulated very strictly, to avoid free coproheme within the cell. This is reflected in the determined affinities of coproheme to apo-*Lm*ChdC and apo-*Lm*CpfC (Fig. 3) [19]. The fact that *Lm*CpfC binds coproheme with a very similar but slightly higher affinity than *Lm*ChdC is most probably a guarantee that no free coproheme can escape into the cell. Therefore, trafficking of coproheme to ChdC is a crucial process, which will be investigated in future, as any unregulated, free coproheme present within the cell would damage the cell.

Mechanistic details on ferrous iron insertion into a porphyrin macrocycle and release of the product have been in the focus of research for several decades. A distorted porphyrin was proposed to be a relevant intermediate for the iron insertion process. Initial considerations derived from structures with the bound inhibitor NMMP, which showed distortion of ~ 35° [10,11,29,30], and the maximal distortion of the porphyrin macrocycle in human ferrochelatase was 12° [31]. Next to porphyrin distortion, a conformational switch in human ferrochelatase was determined upon binding, which also has some mechanistic implications [32]. RR studies revealed porphyrin distortion and showed that a saddled/distorted porphyrin is bound and a flatter, metalated porphyrin is released [33]. The evidence for a distorted porphyrin is given, but the exact degree and whether it is the cause for iron insertion and product release, or whether it occurs as a consequence of the enzymatic reaction is still under discussion. We here show that NMMP binds differently to CpfCs than coproheme and also that coproporphyrin III and coproheme thermodynamically stabilize monomeric *Lm*CpfC to the same degree, implicating highly similar porphyrin interactions with the protein moiety.

In summary, we present a spectroscopic, enzymatic, and structural study of CpfC from *L. monocytogenes*, revealing the catalytically relevant binding orientation of the porphyrin substrate and product. The knowledge of interaction sites of the porphyrin with the protein moiety is the basis for future studies, investigating the governing forces of substrate/product binding, catalytic turnover, and product release. All these factors are crucial within the prokaryotic CPD heme biosynthesis pathway, as heme uptake, degradation, and synthesis have to be tightly regulated as the equilibrium of this important cofactor is crucial to the viability of an organism [34,35], and specific inhibition would be potentially interesting to combat pathogens like *L. monocytogenes*. Recently, the interaction and regulatory effect between CpfC, ChdC, and the heme monooxygenase IsdG was shown in *S. aureus* [36], emphasizing the complexity of protein–protein interactions necessary within prokaryotic heme synthesis, uptake, and degradation in Firmicutes.

## Materials and methods

### Cloning, expression, and purification

*Lm*CpfC was amplified from genomic DNA (acquired from ATCC, Old Town Manassas, VA, USA) by PCR with primers (forward: ggacagcaaatgggtcgcggatccatgactaaaaaagtag; reverse: gtggtggtggtggtgctcgag attgctatatttttcccag) having overhangs suitable for Gibson assembly to incorporate the target sequences into the pET21a(+) vector, which was restricted using XhoI and BamHI. Obtained plasmids carrying information for *Lm*CpfC and a C-terminal 6 × His-tag for purification via metal chelate affinity chromatography were transformed into *E. coli* BL21 GOLD cells and cultivated in LB medium supplemented with 1 mm ampicillin. Expression was started by induction with 0.5 mm IPTG at an OD_600_ of about 0.4–0.6. Cells were further cultivated overnight at 16 °C and harvested by centrifugation (2430 ***g***). Cells were lysed by sonication, and the lysate was applied to a HisTrap column and ultimately eluted using an imidazole gradient (0–500 mm). Samples were buffer exchanged using PD-10 columns and stored in 1 × PBS buffer with 100 mm NaCl, pH 7.4 at −80°C. Reconstitution of *Lm*CpfC for crystallization with coproheme was performed by the addition of a 1.2 fold molar excess, and the complex was purified by size-exclusion chromatography (SEC) (Superdex 200, 16/600).

### Determination of the oligomeric assembly in solution

SEC-MALS was performed to determine the molar mass and oligomeric state of apo-, coproporphyrin III-, and coproheme-*Lm*CpfC. HPLC (Shimadzu prominence LC20, Korneuburg, Austria) was equipped with MALS (WYATT Heleos Dawn8 + plus QELS; software astra 6, Dernbach, Germany), refractive index detector (RID-10A; Shimadzu), and a diode array detector (SPD-M20A; Shimadzu). The column (Superdex 200 10/300 GL; GE Healthcare, Vienna, Austria) had a particle size of 13 μm and was equilibrated with 1 × PBS plus 200 mm NaCl. Flow rate was 0.75 mL·min^−1^, and the injected protein amount was 50 μg. Proper performance of molar mass calculation by MALS was verified by the determination of a sample of bovine serum albumin. This method was performed similarly as previously described [37].

### Crystallization

Apo-*Lm*CpfC (9.5 mg·mL^−1^) crystallized within 2 days in condition D12 (16% w/v PEG 8000, 20% v/v Glycerol, 0.04 M KH_2_PO_4_) of the JCSG + HT96 screen (Molecular Dimensions, Sheffield, UK), which was already a suitable condition for cryocooling. Drops for all experiments were set using the Mosquito LCP (TTP Labtech, Melbourn, UK) on SWISSCI MRC three-well plates, testing three different ratios of protein (150 nL) to crystallization solution (100, 150, 200 nL). Coproheme-*Lm*CpfC (9.5 mg·mL^−1^) crystals were obtained in condition H11 (0.1 m BIS-TRIS pH 5.5, 25% PEG3350, 0.2 m MgCl_2_) of the JCSG + screen by setting up crystals with extra 100 nL of a seeding solution (1 : 1000 dilution of harvested apo-*Lm*CpfC crystals).

### X-ray data collection structure determination and refinement

Data for both datasets were collected at beamline I03 of the Diamond Light Source (Harvell Science and Innovation Campus, Didcot, UK) at 100 K using a DECTRIS EIGER2 X 16M detector. The XDSAPP pipeline was used for processing of the obtained datasets. The phase problem was solved initially for the apo-*Lm*CpfC structure by molecular replacement using Phaser-MR [38] taking the pdb structure 2HK6, from *Bs*CpfC. The coproheme-*Lm*CpfC dataset was phased using the pdb structure of the solved apo-*Lm*CpfC. AUTOBUILD [39] was used to generate an initial model, which was further improved by iterative cycles of manual model building using COOT [40] and maximum likelihood refinement using PHENIX-refine [41]. PHENIX-refine converted intensities into amplitudes using the French and Wilson algorithm [42]. Restraints for coproheme (Ligand ID RM9) were generated using eLBOW, taking an sdf file as input and applying the final-geometry option. Final stages of refinement included Translation Liberation Screw (TLS) parameters, isotropic *B*-factor model, automated addition of hydrogens and water molecules, optimization of X-ray/ADP weight, and optimization of X-ray/stereochemistry weight. The model was validated with MolProbity [43]. Figures were prepared with PyMOL (http://www.pymol.org).

### Substrate channel calculation

CAVER [17] was used to detect putative channels to the coproporphyrin III/coproheme binding site of apo- and coproheme-*Lm*CpfC. For calculation of the characteristics of the channels, the coproheme iron of coproheme-*Lm*CpfC was set as a starting point. Channels were calculated with the following settings: minimum probe radius: 0.9 Å; shell depth: 10 Å; shell radius: 9 Å; clustering threshold: 3.5; number of approximating balls: 12; input atoms: 20 amino acids (without other components present in the respective structural models). This method was performed similarly as previously described [37].

### Determination of coproheme and coproporphyrin III binding constants

Time-resolved binding of coproheme and coproporphyrin III to apo-*Lm*CpfC was monitored using a stopped-flow apparatus equipped with a diode array detector (model SX-18MV; Applied Photophysics, Leatherhead, UK), in the conventional mode. The optical quartz cell with a path length of 10 mm had a volume of 20 μL. The fastest mixing time was between 1.0 and 1.5 ms. All measurements were performed at 25°C. Typically, the concentration of the respective ligand was kept constant in the cell at 1 μm and *Lm*CpfC was present in excess (5–10 μm for coproheme binding; 10–35 μm for coproporphyrin III), to ensure pure spectral species of the ligand bound proteins. Experiments were carried out in 50 mm HEPES buffer, pH 7.0. In order to determine the second-order rate constant, *k*_on_, and the first-order rate constant *k*_off_, *k*_obs_ values were plotted *versus Lm*CpfC concentration; *K*_D_ was calculated by *k*_off_/*k*_on_. *k*_obs_ was determined by recording time traces (in triplicates) at a single wavelength (393 nm for coproheme binding; 410 nm for coproporphyrin III binding). Resulting time traces were fitted single exponentially for coproheme binding and double exponentially for coproporphyrin III binding. This method was performed similarly as previously described [19].

Competition binding assays (in 50 mm HEPES, pH 7.0) of 5 μm coproheme bound to *Lm*ChdC or *Lm*CpfC were conducted by preforming one coproheme–protein complex and increasing the concentration of the other protein by stepwise titration (0–25 μm). UV-vis spectra were recorded using a Cary 60 (Agilent, Santa Clara, CA, USA) scanning spectrophotometer. *Lm*ChdC was expressed and purified as described previously [21]. Absorbance at 375 nm was plotted *versus* the protein concentration and fitted to a hyperbola function to derive *K*_D_ values.

### Enzymatic activity

Insertion of ferrous iron into coproporphyrin III was monitored spectrophotometrically using a Cary 60 (Agilent) spectrophotometer, equipped with a pulsed lamp for photosensitive samples, in the scanning mode (700–250 nm), in order to see the spectral changes of coproporphyrin III loaded *Lm*CpfC to coproheme-*Lm*CpfC upon addition of ferrous iron; the protein was present in excess to ensure complete substrate binding. The reaction was carried out anaerobically under argon flow and in the dark. From this setup, an aliquot was taken from the cuvette and analyzed by mass spectrometry as follows.

The sample mixture (5.0 μL each) was analyzed using a Dionex Ultimate 3000 system directly linked to a QTOF mass spectrometer (maXis 4G ETD; Bruker, Rheinstetten, Germany) equipped with the standard ESI source in the positive ion mode. MS scans were recorded within a range from 400 to 3800 *m/z*, and the instrument was tuned to detect both the rather small free heme derivatives and intact proteins in a single run. Instrument calibration was performed using ESIcalibration mixture (Agilent). For separation of the analytes, a Thermo ProSwift™ RP-4H analytical separation column (250 × 0.200 mm) was used. A gradient from 99% solvent A and 1% solvent B (solvent A: 0.05% trifluoroacetic acid, B: 80.00% acetonitrile and 20% solvent A) to 65% B in 11 min was applied, followed by a 2-min gradient from 65% B to 95% B, at a flow rate of 8 μL·min^−1^ and at 65°C. A blank run (5.0 μL H_2_O) was performed after each sample to minimize carry-over effects. This method was performed similarly as previously described [3].

Michaelis–Menten parameters of ferrochelatase activity of *Lm*CpfC for ferrous iron were determined by varying the Fe^2+^ concentration, analogous to a previously described assay [15]. In short, the depletion of absorbance at 392 nm was followed in the dark under anaerobic conditions to derive initial velocities. The enzyme concentration was 100 nm, and Fe^2+^ concentration was varied between 0.1 and 5.0 μm. The concentration of coproporphyrin III was kept constant at 5 μm.

Reaction of hydrogen peroxide with coproheme-*Lm*CpfC (2 μm) was tested using H_2_O_2_ concentrations ranging from 10 μm to 10 mm in 50 mm HEPES buffer, pH 7.0. The reaction was followed spectroscopically and on a Clark-type electrode to monitor potential oxygen generation. No other effect than heme bleaching was observed whatsoever.

### Spectroscopy of *Lm*CpfC

UV-vis absorption spectroscopy was carried out using the Cary 60 (Agilent), equipped with a pulsed lamp for photosensitive samples, scanning photometer between 700 and 250 nm. The substrate coproporphyrin III was titrated, starting with subequimolar concentrations to *Lm*CpfC, in order to obtain a spectrum representing the pure coproporphyrin III loaded ferrochelatase. Vice versa, free coproporphyrin was recorded first and apo-*Lm*CpfC was titrated to a large excess. The same procedure was performed with the product coproheme. This method was performed similarly as previously described [3].

For determination of the spin state of ferric iron of the product-bound form, EPR was performed on a Bruker EMX continuous-wave (cw) spectrometer, operating at X-band (9 GHz) frequencies. The instrument was equipped with a high-sensitivity resonator and an Oxford Instruments ESR900 helium cryostat for low-temperature measurements. Spectra were recorded under nonsaturating conditions using 2 mW microwave power, 100 kHz modulation frequency, 1 mT modulation amplitude, and 40-ms conversion time, 40-ms time constant and 2048 points. Samples of recombinant coproheme-*Lm*CpfC (100 μL of 100 μm) were prepared in 100 mm HEPES buffer, pH 7.0, and 20% glycerol, transferred into Wilmad quartz tubes (3 mm inner diameter), and flash-frozen in liquid nitrogen. In order to remove O_2_, the tubes were flushed with argon, while the sample was kept frozen on dry ice. Measurements were performed at 10 K. The spectra were simulated with the EasySpin toolbox for Matlab [44] and consist of a weighted sum of simulations of the individual HS and low-spin species. The rhombicity was obtained from ${\text{g}}_{\text{x}}^{\text{eff}}$ and ${\text{g}}_{\text{y}}^{\text{eff}}$ and the relative intensities were calculated on the basis of the simulations, following the procedure of Aasa and Vanngard to account for the different integral intensity per unit spin of species that display different effective g-values (as found in potential low-spin and HS centers) [45,46]. This method was performed similarly as previously described [13].

### Thermal stability of *Lm*CpfC

Differential scanning calorimetric measurements were performed using an automated PEAQ-DSC (Malvern Panalytical, Malvern, UK) with a cell volume of 130 μL. The measurements were controlled by the integrated microcal-peaq-dsc software (Malvern, UK), and the instrument was equipped with an autosampler for 96-well plates. Samples were analyzed using a programmed heating scan rate of 60°C·h^−1^ over a temperature range from 20 to 100°C, and cell pressure was ~ 60 psi (4.136 bar). Thermograms were corrected for buffer baseline and protein concentration; 14 μm of apo-, coproporphyrin III-, or coproheme-*Lm*CpfC in 50 mm HEPES buffer, pH 7.0, was used for each measurement. For data analysis and conversion, the integrated analysis software was used. Heat capacity (*C*_p_) was expressed in kJ mol^−1^·K^−1^. Data points were fitted to non-two-state equilibrium-unfolding models by the Lavenberg/Marquardt nonlinear least squares method. This method was performed similarly as previously described [19].

### Phylogenetic analysis

A selection of 28 ferrochelatase sequences was collected from public databases (Uniprot, NCBI). First, multiple sequence alignments were constructed using MUSCLE [47] with following parameters: gap penalties −2,9 and gap extension 0; hydrophobicity multiplier 1.2; max iterations 8. From these sequence alignments, a phylogenetic tree of CpfC and PpfC proteins was reconstructed with the Maximum Likelihood algorithm using the Jones-Taylor-Thornton model, the γ parameter set to 3, and with 1000 bootstrap replications; complete deletion was used for gaps/missing data treatment. All tools for sequence alignments and phylogenetic tree reconstruction were embedded in the MEGA5 package [48]. The phylogenetic tree was drawn with FigTree v1.4 (http://tree.bio.ed.ac.uk/software/figtree/). This method was performed similarly as previously described [49].

## Figures and Tables

**Table 1:** Statistics of data collection and structure refinement of apo- and coproheme-*Lm*CpfC (values for the highest resolution shell are presented in brackets).

	Apo-*Lm*CpfC (6RWV)	Coproheme-*Lm*CpfC (6SV3)
Wavelength (Å)	0.9800	0.9763
Resolution range	50.09–1.639 (1.698–1.639)	36.5–1.64 (1.699–1.64)
Space group	P 1 21 1	P 1 21 1
Unit cell	48.30 76.72 52.26 90 106.56 90	37.47 68.14 62.91 90 103.06 90
Total reflections	89 584 (8829)	71 382 (6465)
Unique reflections	44 834 (4416)	37 446 (3635)
Multiplicity	2.0 (2.0)	1.9 (1.8)
Completeness (%)	99.84 (99.10)	99.04 (96.68)
Mean *I*/sigma(*I*)	6.50 (1.09)	12.36 (1.15)
Wilson *B*-factor	18.08	24.29
*R*-merge	0.06407 (0.6822)	0.04989 (0.607)
*R*-meas	0.09061 (0.9648)	0.07055 (0.8584)
*R*-pim	0.06407 (0.6822)	0.04989 (0.607)
CC1/2	0.997 (0.435)	0.997 (0.561)
CC*	0.999 (0.779)	0.999 (0.848)
Reflections used in refinement	44 797 (4416)	37 444 (3635)
Reflections used for *R*-free	2239 (228)	1822 (189)
*R*-work	0.1760 (0.2749)	0.1713 (0.3885)
*R*-free	0.2013 (0.3221)	0.2014 (0.4914)
CC(work)	0.954 (0.741)	0.969 (0.721)
CC(free)	0.948 (0.704)	0.954 (0.576)
Number of nonhydrogen atoms	2887	2928
Macromolecules	2565	2544
Ligands	54	61
Solvent	268	309
Protein residues	310	309
RMS(bonds)	0.009	0.009
RMS(angles)	0.93	0.90
Ramachandran favored (%)	97.40	97.07
Ramachandran allowed (%)	2.60	2.93
Ramachandran outliers (%)	0.00	0.00
Rotamer outliers (%)	0.37	0.74
Clashscore	3.69	6.69
Average *B*-factor	22.36	25.84
Macromolecules	20.19	24.22
Ligands	54.24	29.98
Solvent	36.76	37.75
Number of TLS groups	1	1

## Abbreviations

5c: 5-coordinated
6c: 6-coordinated
*Bs*: *Bacillus subtilis*
CgoX: coproporphyrinogen oxidase
ChdC: coproheme decarboxylase
CPD: coproporphyrin-dependent
CpfC: coproporphyrin ferrochelatase
cw: continuous-wave
DSC: differential scanning calorimetry
dSDP: 2,4-disulfonic acid deuteroporphyrin IX
HS: high-spin
*Lm*: *Listeria* monocytogenes
MALS: multi-angle light scattering
NMMP: *N*-methylmesoprophyrin
PgoX: protoporphyrinogen oxidase
PPD: protoporphyrin-dependent
PpfC: protoporphyrin ferrochelatase
RR: resonance Raman
*Sa*: *Staphylococcus* aureus
SEC: size-exclusion chromatography
